# A novel mutation in the *proteoglycan 4* gene causing CACP syndrome: two sisters report

**DOI:** 10.1186/s12969-023-00793-z

**Published:** 2023-01-24

**Authors:** İlknur Bağrul, Serdar Ceylaner, Yasemin Tasci Yildiz, Serife Tuncez, Elif Arslanoglu Aydin, Esra Bağlan, Semanur Ozdel, Mehmet Bülbül

**Affiliations:** 1 Department of Pediatric Rheumatology, Dr Sami Ulus Maternity and Child Health and Diseases Training and Research Hospital, Ankara, Turkey; 2 Department of Genetics, Intergen Genetics Centre, Ankara, Turkey; 3 Department of Pediatric Radiology, Dr Sami Ulus Maternity and Child Health and Diseases Training and Research Hospital, Ankara, Turkey

**Keywords:** Arthropathy, Camptodactyly, Coxa vara, PRG4 mutation

## Abstract

**Background:**

Camptodactyly-arthropathy-coxa vara-pericarditis (CACP) syndrome, caused by biallelic pathogenic mutations in the *proteoglycan 4* (*PRG4*) gene, is characterized by early-onset camptodactyly, noninflammatory arthropathy, coxa vara deformity, and rarely, pericardial effusion. This syndrome can mimic juvenile idiopathic arthritis. CACP syndrome is caused by mutations in the *proteoglycan 4* (*PRG4*) gene. To date, only 36 pathogenic mutations have been reported in this gene, but none have been reported from Azerbaijan.

**Case presentation:**

Herein, we report two siblings presented with chronic polyarthritis, had a prior diagnosis of juvenile idiopathic arthritis, but was subsequently diagnosed as CACP syndrome with novel mutation in the *PRG4* gene.

**Conclusion:**

Our report expands the knowledge of PRG4 mutations, which will aid in CACP patient counseling.

## Background

Camptodactyly-arthropathy-coxa vara-pericarditis syndrome (CACP, OMIM208250) is a rare autosomal recessive disease caused by *proteoglycan 4* (*PRG4*) gene mutation [1, 2]. The disease is characterized by congenital or early onset camptodactyly, and non-inflammatory arthropathy, progressive coxa vara deformity, and sterile pericardial effusion/pericarditis [3]. CACP syndrome should be considered in the presence of symmetrical arthropathy, camptodactyly, and normal levels of inflammatory markers in large joints. Arthropathy in CACP syndrome patients primarily involves the large joints, such as the elbows, hips, knees, and ankles [4]. Non-inflammatory pericarditis has been reported in approximately 33% of published CACP syndrome cases. Pericarditis is usually mild or self-limiting, but in rare cases it can be severe. Coxa vara was noted in 50–100% of CACP syndrome cases [4, 5]. In this regard, we report two Azerbaijani siblings presented with chronic polyarthritis who was diagnosed initially juvenile idiopathic arthritis, and subsequently diagnosed with CACP syndrome with a novel mutation in the PRG4 gene is reported.

## Case presentations

### Patient 1

A 15-year-old Azerbaijani female presented to our pediatric rheumatology department with bilateral swelling, pain, and limited movement of the elbows, wrists, fingers, knees, and distal interphalangeal (DIP) joints, and bilateral hip joint pain. Limitation of bilateral finger movements started when she was age 7 months, at which time her family did not notice any significant swelling. Swelling and pain in the elbows, knees, ankles, and toes began when she was age 5 years. She is the first child of healthy first-degree consanguineous parents. She has a 13-year-old sister with similar joint complaints. The patient has a multi-year history of recurrent hospitalizations due to joint complaints. During this period the patient was diagnosed juvenile idiopathic arthritis.

**Fig. 1:**
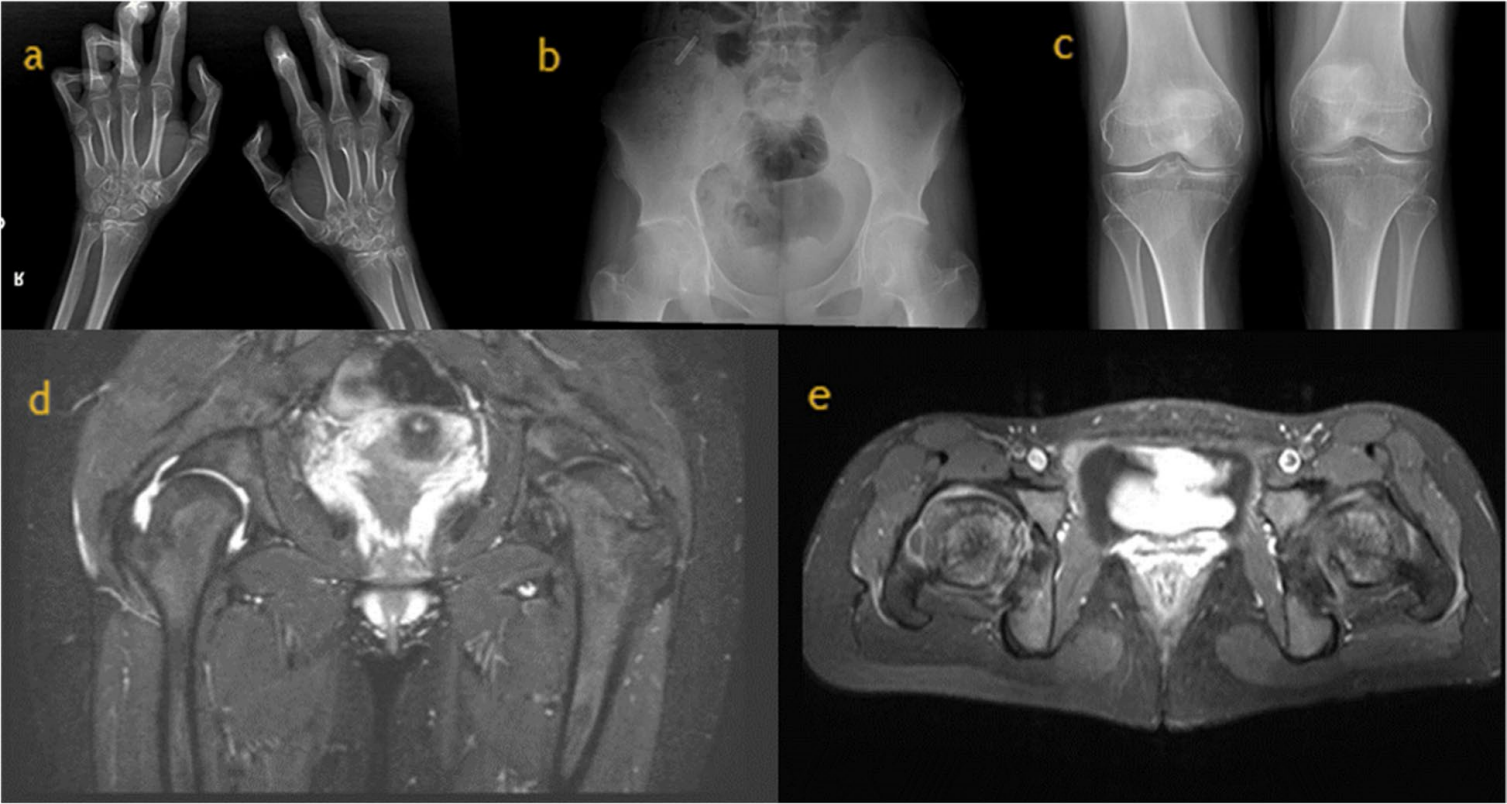
Radiological evaluation of “Patient 1”. **a** Depicts narrowing of the wrist joint spaces with periarticular loss of bone dansity. Contractures were observed in the distal interphalangeal joints. Subchondral cysts were observed in the distal epiphysis of both radius and ulna. The 5th metacarpal bone of the left hand was short. **b** Bilateral hip joint spaces are markedly narrowed. Bilateral coxa vara deformity was observed. Bilateral femoral heads were flat, femoral necks were short. **c** The joint space was narrowed in both knees. **d** Chronic degenerative changes were observed in both hip joints. Coxa vara deformity, narrowing of the joint spaces, millimetric subchondral cysts on the femoral heads were observed. The changes were more prominent on the left side. Coxa magna deformity was detected in the left femur. **e** Fluid increase in the joint space and contrast enhancement consistent with synovitis were observed on the left side

Upon physical examination, there was swelling, movement limitation, and contractures of both knees, wrists, elbows, shoulders, hips, and ankles, flexion limitation of the 3rd and 4th right proximal interphalangeal (PIP) joints, flexion contracture of the 3rd and 4th PIP joints of the right hand and 4th PIP joint of the left hand, bilateral overlapping toe deformity, and bilateral axillary region squamous rashes. Other systemic examinations were normal. Radiological evaluation of patient 1 was given in Fig. 1.

### Patient 2

Patient 1’s 13-year-old sister had pain, limitation of movement, and bilateral deformities in her hands, feet, and shoulder joints. Since infancy she had bilateral limitation of finger movement, and at age 6 years bilateral pain and deformities began in her elbows, knees, ankles, and toes. Upon physical examination there was bilateral limited range of motion and contractures in the knees, wrists, elbows, and ankles. Hip and shoulder movements were painful. There were flexion contractures in the 3rd and 4th PIP joints of the right hand, and flexion contractures in the 4th PIP joints of the left hand. Other systemic examinations were normal. No other family member had similar complaints. Radiological evaluation of patient 2 was given in Fig. 1.

Laboratory test results in both patients were as follows: Complete blood count and biochemical tests: normal; acute phase reactants: negative; antinuclear antibody, human leukocyte antigen-b27, anti-cyclic citrullinated peptide antibody, and rheumatoid factor: negative; complement tests and immunoglobulins: normal. Uveitis was not observed in patient 1, whereas bilateral asymptomatic, non-granulomatous, anterior uveitis was noted in patient 2.

**Fig. 2:**
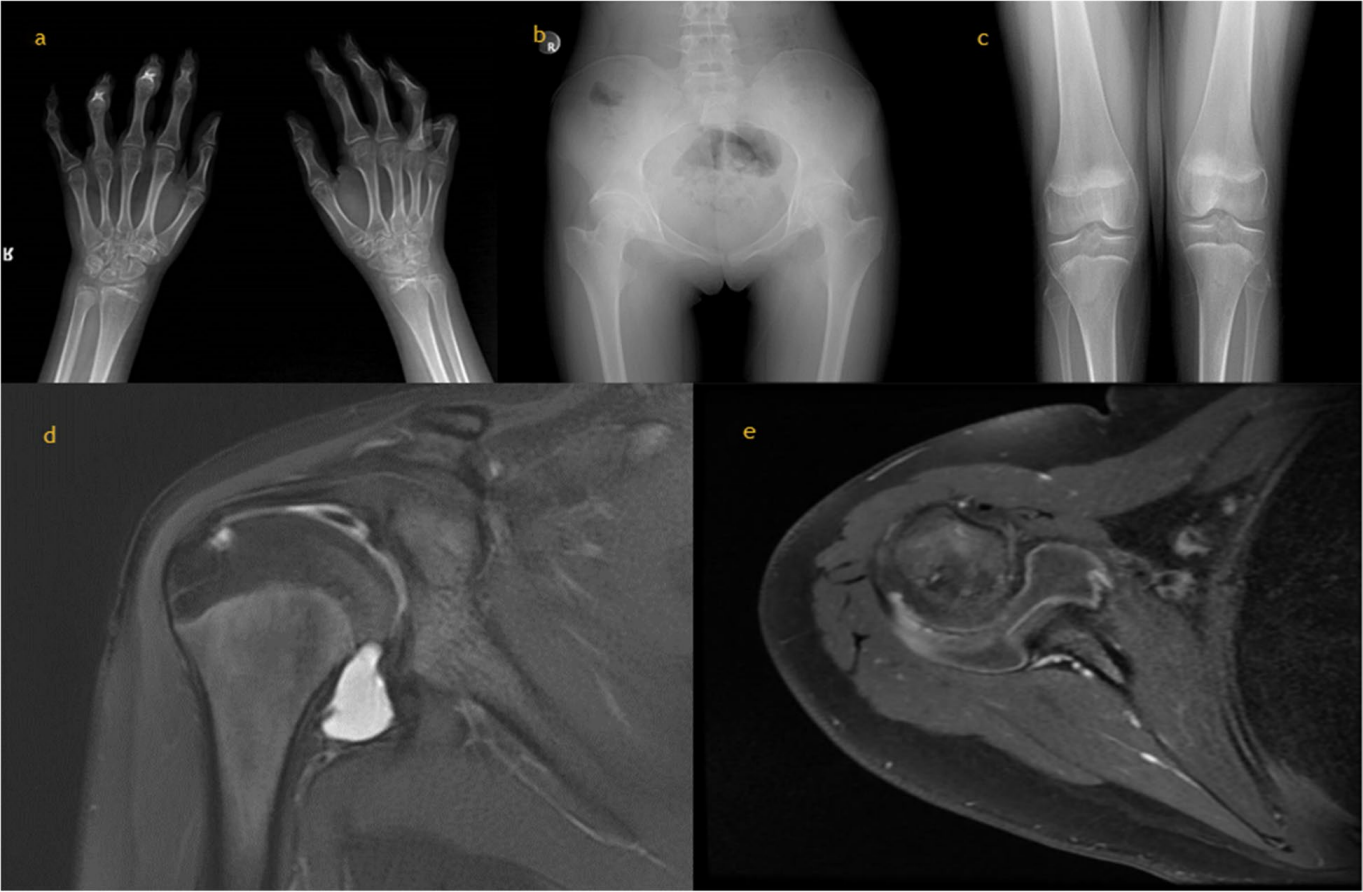
Radiological evaluation of “Patient 2”. **a** Skeletal survey reveals heterogeous lucensies of the carpal bones, distal radial and ulnar epiphysis. The joint spaces in the wrists and proximal and distal interphalangeal joints were markedly narrow. Periarticular bone resorption in MCP, PIP, DIP joints and carpal bones were noted. **b** Both hip joint spaces were narrow and coxa vara deformity was present. **c** Periarticular bone resorption adjacent to knee joints were observed along with joint space narrowing. Lucensies were noted on the proximal diaphysis of left tibia. **d** Millimetric subchondral cysts on the humeral head and dense fluid in the joint space and subscapular bursa. **e** The synovium enhanced after contrast administration consistent with synovitis

Skeletal survey of patient 1 (Fig. 1) revealed narrowing of the elbow and wrist joint spaces with periarticular osteoporosis. Contractures were observed in the distal interphalangeal joints. Subchondral cysts were observed in the distal epiphysis of both radius and ulna. The 5th metacarpal bone of the left hand was short. Bilateral hip joint spaces are markedly narrowed. Bilateral coxa vara deformity was observed. Bilateral femoral heads were flat, femoral necks were short. The joint space was narrowed in both knees. Contrast enhanced magnetic resonance imaging (MRI) of bilateral hip joints of the patient 1 was performed: ‘Chronic degenerative changes were observed in both hip joints. Coxa vara deformity, narrowing of the joint spaces, millimetric subchondral cysts on the femoral heads were observed. The changes were more prominent on the left side. Coxa magna deformity was detected in the left femur. Fluid increase in the joint space and contrast enhancement consistent with synovitis were observed on the left side. Millimetric cysts with peripheral enhancement were observed in the left iliac bone. Bilateral contrast enhancement on the greater trochanter of femur were noted. Skeletal survey of patient 2 (Fig. 2) revealed heterogeous lucensies of the right proximal humeral epiphysis, carpal bones, distal radial and ulnar epiphysis. The joint spaces in the wrists and proximal and distal interpharyngeal joints were markedly narrow. Periarticular bone resorption in MCP, PIP, DIP joints and carpal bones were noted. Similarly, periarticular bone resorption adjacent to knee and ankle joints were observed along with joint space narrowing. Lucensies were noted on the proximal diaphysis of left tibia. Both hip joint spaces were narrow and coxa vara deformity was present. Contrast enhanced MRI of right shoulder of the patient 2 was performed. There were millimetric subchondral cysts on the humeral head. There was dense fluid in the joint space and subscapular bursa which has synovial enhancement after contrast administration consistent with synovitis.

Both patients were referred to the genetics department. Whole-exome sequencing was performed in both patients, which showed the existence of (to the best of our knowledge) a novel pathogenic (NM_005807.6):c.3445A > T(p.Lys1149Ter) homozygous mutation in the *PRG4* gene associated with CACP syndrome. The patients were diagnosed with CACP syndrome and followed-up. Physical therapy was prescribed for both patients.

A search of the English-language literature for original articles, case reports, and series using the keywords camptodactyly-arthropathy-coxa vara-pericarditis syndrome and various databases (i.e. PubMed/Medline, Scopus, and Embase) was performed. Reports of CACP syndrome with proven genetic mutation published before October 2022 were included in the literature review. In total, 13 reports that included 106 CACP cases with proven genetic mutations were found. In all, 36 pathogenic mutations were reported. A summary of the clinical findings of these cases is presented in the Table 1. All 106 of the published patients had arthritis in the large joints, camptodactyly was present in 103 of the patients, coxa vara deformity was present in 100 patients, and pericarditis was present in 14. In all, 34 of the 54 represented families had consanguineous parents. There were also 31 point mutations reported in ClinVar database and 22 of them were frameshifts and 9 of them were non-sense variants as in our case.

**Table 1:** Literature review of mutation-proven CACP syndrome cases

Publication	Number of patients	Mutation(s) reported	Molecular defect	Clinical properties							Date of Publication
				Consanguineous parents	Age at diagnosis-(years)	Camptodactyly	Arthropathy in large joints	Coxa vara deformity	Pericarditis	Patient nationality	
Alazami et al. [3]	7	4	c.3139_3140delAA c.4078A > T c.923_924delAA c.3125_3128delGAGT c.3276_3277delAA	3/4	1.5–8	7/7	7/7	5/7	0/8	Arabian	2006
Peters et al. [4]	1	1	c.1290del	1/1	10	1/1	1/1	1/1	1/1	Turkish	2016
Yilmaz et al. [5]	35	10	c.1194delC c.3917_3934del c.3276_3277delAA c.4101C > G c.1192delC c.1911delT c.2215A > T c.1910_1911delCT c.2837_2838delAA c.849delA	10/11	1–52	35/35	35/35	35/35	3/35	Turkish, Northern Iraqi	2018
Marcelino et al. [6]	8	5	c.2805del5 c.3240del7 c.3023del2 c.3690del5 c.4190CC → AG	4/4	2–13	8/8	8/8	7/8	2/8	Arabian Canadian	1999
Albuhairan and Al-Mayouf et al. [7]	22 (15 patients previously published)	5 (new)	c.923_924delAA c.3125_3128delGAGT c.3139_3140delAA c.3276_3277delAA c.4078A > T	10/15	1–14	22/22	22/22	22/22	2/22	Arabian	2013
Patil et al. [8]	2	1	c.884_885delAG	0/1	9–13	2/2	2/2	2/2	2/2	Indian	2016
Kisla Ekinci et al. [9]	3	–	–	2/2	3–8	3/3	3/3	2/3	0/3	Turkish	2021
Basit et al. [10]	6	1	c.2816_2817delAA	1/1	NA	6/6	6/6	6/6	0/6	Pakistani	2011
Akawi et al. [11]	4	1	c.1320dupC	1/1	5–12	4/4	4/4	4/4	0/4	Arabian	2012
Mannurita et al. [12]	13	5	c.1982_1983delCT c.2153delA c.2754_2758delGACAA c.3636 + 3A > G c.3648C > A	1/10	0–15	10/13	13/13	11/13	4/13	European cohort (Italian, Albanian, and Dutch)	2014
Nandagopalan et al. [13]	3	2	c.2645_2646delGA c.2883_2886delAAGA	0/2	5–13	3/3	3/3	3/3	0/3	Indian	2014
Johnson et al. [14]	2	1	chr1:186275834dupT	1/2	12–13	2/2	2/2	2/2	0/2	Indian	2021
Total	106	36	–	34/54	–	103/106	106/106	100/106	14/106	–	–

### Molecular method

DNA isolation was performed using automated magnetic seperator. Exome enrichment was performed using Twist Comprehensive Human Exome kit according to manufacturers instructions. Prepared library was sequenced on MGI DNBSEQ-G400 at 80-100X on-target depth with 150 bp paired-end sequencing at Intergen Genetic Diagnostic Centre Ankara/Turkey. Bioinformatics analyses were performed using in-house developed workflow derived from GATK best practices. Raw reads were cleaned from MGI adapter sequences using cutadapt. Alignment to GRCh38 was done using BWA-MEM 0.7.17. Variant calling was performed using GATK HaplotypeCaller and low quality variants were eliminated based on strand bias, read depth and call quality parameters and other related parameters. Copy number variations were inferred using GATK GermlineCNVCaller. ACMG criteria are used to evaluate whether these changes are the cause of the disease. *PRG4* (NM_005807.6):c.3445A > T(p.Lys1149Ter) homozygous pathogenic variant was detected. As null variants are pathogenic in this gene and there are some other null variants were reported before and after this codon, this variant was classified as pathogenic. This is a novel variant.

## Discussion

CACP is a rare autosomal recessive inherited disorder previously associated with alterations in the *PRG4* gene coding for lubricin. This gene, located on chr 1q25-q31, contains 12 exons spanning 18 kb and is responsible for the production of the glycoprotein lubricin [6]. To date, 36 pathogenic *PRG4* mutations have been reported, but to date none have been reported from Azerbaijan. The aim of presenting the 2 presented cases was to add a novel *PRG4* mutation in the 2 sisters with CACP syndrome to the literature. An additional aim was to highlight the need to improve awareness of CACP syndrome.

The *PRG4* gene is the only gene known to be associated with CACP syndrome. Among the products of full-length *PRG4* transcripts is a secreted glycoprotein (lubricin) of 1404 amino acids, which is secreted by synovial fibroblasts and articular chondrocytes. Lubricin is found in many tissues, including synovial fluid, the surface of cartilage, and tendons. In addition to its major lubricating role in joints, it regulates cell growth and protects the cartilage surface from friction-induced damage [6, 15, 16]. Production of mutant lubricin inhibits the normal exchange of nutrients and waste products, and causes protein deposition, cell adhesion, and synovial hyperplasia that invades the articular cartilage surface. Synovial fluid helps reduce the pressure applied to a joint surface during movement [17]. An earlier study on the role of lubricin in synovial fluid suggests that lubricin-deficient joint fluid in CACP syndrome patients performs this function less well than joint fluid not deficient in lubricin [18].

The main clinical features of CACP syndrome are camptodactyly, non-inflammatory arthropathy, progressive coxa vara deformity, and pericardial effusion. The disease often presents as camptodactyly in early childhood. Camptodactyly is the first symptom in patients with CACP and tends to be symmetrical. It usually occurs during the first days or weeks of life [2]. Large joints are usually normal during the neonatal period. Symptoms appear with age, usually within the first 12 months. In early childhood the first affected large joints are the wrists. Nearly all CACP syndrome patients have extensive joint involvement, including bilateral arthropathy resulting in swelling, limitation of motion, and flexion contractures, with the absence of any inflammatory signs. Elbow, wrist, and knee joints are affected in all patients, whereas only the ankle joints are affected in some patients [2, 3, 19].

Radiographically, the most common findings include coxa vara deformity, osteopenia, osteoporosis, increased joint space, and a wide and short femoral neck. Spine involvement also occurs, with the exception of the cervical spine. Coxa vara is present in 50–100% of cases and is progressive [5, 7, 20]. Non-inflammatory pericarditis probably occurs due to the lack of surface lubrication between the visceral and parietal pericardium layers, and is reported in 6–30% of patients with CACP syndrome. Pericarditis has wide clinical variability, from a self-limiting course to constrictive pericarditis requiring surgical intervention. Recurrent episodes of pericarditis can cause constrictive pericarditis, which is indicated based on signs of heart failure [4, 8].

CACP syndrome can be confused with inflammatory arthropathies in many ways. CACP syndrome patients are often mistakenly diagnosed with inflammatory arthritis due to the early onset of progressive arthritis and multi-joint involvement, and then receive anti-inflammatory therapy, which does not provide any benefit in the long-term. In a cohort of 35 CACP syndrome patients reported by Yılmaz et al. [5] mean age at diagnosis was 12 years; therefore, clinical differentiation of CACP syndrome and juvenile idiopathic arthritis (JIA) is essential. In patients with CACP syndrome, inflammatory parameters are normal, in contrast to JIA patients. Flattening of the metacarpal and phalangeal heads, periarticular osteopenia, osteoporosis, a short femoral neck, short iliac wings, coxa vara with wide and irregular femoral heads, and large intraosseous fluid-filled cysts are radiological findings considered to be specific to CACP syndrome [20, 21]. In addition, consanguineous parents, family members with a similar disease, early-onset camptodactyly, bilateral arthropathy, and blood or joint fluid samples with normal levels of inflammatory markers can be used to differentiate CACP syndrome from JIA.

The first case of CACP syndrome with evidence of *PRG4* mutation was reported in 1999 by Marcelino et al. [6]. Since identification of *PRG4* as the causative gene for CACP syndrome, 36 *PRG4* mutations have been reported. Mutation-proven cases of CACP syndrome reported to date and their clinical characteristics are given in the Table 1.

In 1986 Bulutlar et al. [19] reported the first cases of CACP syndrome from Turkey—4 sisters with consanguineous parents. Mutation-proven cases of CACP syndrome from Turkey were subsequently reported in 2 other studies. In 2018 Yilmazlar et al. [5] reported 10 new mutations in 35 CACP syndrome patients from 11 families, of which 10 had consanguineous parents (to date, the largest cohort in the literature). All the patients had camptodactyly, arthritis of the large joints, and coxa vara deformity. Pericarditis was noted in only 3 (8%) of the patients. Of the 35 patients, 16 were misdiagnosed as JIA and received inflammatory treatment. Another study from Turkey included 3 CACP syndrome cases from 2 consanguineous families. Among the 3 patients, 2 were misdiagnosed as JIA and received anti-inflammatory therapy [9].

There is no effective treatment for arthropathy in children with CACP syndrome. CACP syndrome patients do not respond to treatment with anti-inflammatory medication. Al-Mayouf et al. [22] investigated the efficacy of Yttrium-90 radiosynovectomy in 6 children with CACP syndrome. Although this procedure was described as safe and well tolerated, their study did not show any benefit for arthropathy. Calcium and vitamin D supplementation can be beneficial for the treatment of osteopenia and osteoporosis [23, 24]. Physiotherapy and surgical therapy might be beneficial for joint deformities, and results in pain relief and improvement of function [25]. There are indications that intra-articular injection of recombinant lubricin can prevent synovial thickening and cartilage degeneration due to lubricin deficiency. Rhee et al. showed that in vitro recombinant lubricin inhibits protein aggregation on the cartilage surface and inhibits adhesion-induced cell growth of synoviocytes in lubricin-mutant mice [17]. Another study evaluated the effect of a purified preparation of recombinant lubricin in rats with osteoarthritis lubricin, which had a significant disease-modifying and chondro-protective effect on the progression of osteoarthritis [26]. Moreover, there exists the need to increase awareness of CACP syndrome and to identify an effective treatment focused on the lubrication of joint spaces. In the future the use of recombinant lubricin might be prove to be an option for reducing joint complaints in CACP syndrome patients, but additional clinical studies are needed.

## Conclusion

In this regard, we report two CACP patients, presenting with chronic arthritis during early childhood. Our report expands the knowledge of PRG4 mutations, which will aid in CACP patient counseling. With this report, we wish to highlight the need for early recognition of CACP during differential diagnosis of childhood arthropathies, especially in the presence of camptodactyly.

## Data Availability

Not applicable.

## Abbreviations

CACP: Camptodactyly-arthropathy-coxa vara-pericarditis
PRG4: Proteoglycan 4
DIP: Distal interphalangeal
PIP: Proximal interphalangeal
MCP: Metacarpophalangeal
MRI: Magnetic resonance imaging
JIA: Juvenile idiopathic arthritis
